# Functionalized branched EDOT-terthiophene copolymer films by electropolymerization and post-polymerization “click”-reactions

**DOI:** 10.3762/bjoc.11.39

**Published:** 2015-03-11

**Authors:** Miriam Goll, Adrian Ruff, Erna Muks, Felix Goerigk, Beatrice Omiecienski, Ines Ruff, Rafael C González-Cano, Juan T Lopez Navarrete, M Carmen Ruiz Delgado, Sabine Ludwigs

**Affiliations:** 1 IPOC-Functional Polymers, Institute for Polymer Chemistry, University of Stuttgart, Pfaffenwaldring 55, 70569 Stuttgart, Germany; 2 Thermo Fisher Scientific GmbH, Im Steingrund 4-6, 63303 Dreieich, Germany; 3 Department of Physical Chemistry, University of Málaga, 29071 Málaga, Spain

**Keywords:** band-gap engineering, “click”-chemistry, conducting polymers, electropolymerization, Raman spectroscopy, surface functionalization

## Abstract

The electrocopolymerization of 3,4-ethylenedioxythiophene (EDOT) with the branched thiophene building block 2,2′:3′,2″-terthiophene (3T) is presented as a versatile route to functional polymer films. Comparisons to blend systems of the respective homopolymers PEDOT and P3T by in situ spectroelectrochemistry and Raman spectroscopy prove the successful copolymer formation and the access to tailored redox properties and energy levels. The use of EDOT-N_3_ as co-monomer furthermore allows modifications of the films by polymer analogous reactions. Here, we exemplarily describe the post-functionalization with ionic moieties by 1,3-dipolar cycloaddition (“click”-chemistry) which allows to tune the surface polarity of the copolymer films from water contact angles of 140° down to 40°.

## Introduction

The many different applications of conducting polymers demand for tailored properties, especially the position of the HOMO level and the HOMO–LUMO band gap value are crucial for the applicability in different devices such as organic photovoltaics, organic field effect transistors, organic light emitting diodes or organic electrochromic windows [1–6].

There are different ways to tune HOMO–LUMO band gap values, mostly concerning the modification of the used monomers, for example by a rigidification of the conjugated system [7], the introduction of electron-withdrawing [8] or electron-donating groups [9–10] to the monomers or the increase of the quinoid character [11]. One widely used approach is the introduction of different co-monomers to build up copolymers, e.g., new donor–acceptor low band gap copolymers [12–14]. Among synthetic approaches electropolymerization has gained particular attention, because it allows easy tuning of polymer film properties by modification of the monomers. In addition to electropolymerization of simple conjugated monomers [15] more complex monomers which include different building blocks were presented. Roncali et al. used for example EDOT containing branched thiophene monomers [16–17]. In some of the more complex monomer systems the electropolymerization can be regarded as a crosslinking step [17–18]. Electropolymerization of monomer mixtures is another powerful tool to modify material properties. Among a variety of monomer mixtures including pyrrole and thiophene [19–20], 2,2’-bithiophene and pyrrole [21–22] and dicyanovinylene-substituted cyclopentabithiophene and EDOT [23], we recently presented the copolymerization of EDOT and the branched unit 2,2’:3’,2’’-terthiophene (3T) [24].

Additional functionalities, such as ions, can be introduced either by direct attachment of the functional moieties to the monomers or via precursor monomers which give access to post-polymerization reactions. Ionic groups on conjugated polymers – so-called conjugated polyelectrolytes [25] – are discussed in the context of solubility tuning [26], sensor applications [27], improvement of solar cell performance by usage as hole injection layers [28] or the modification of the surface polarity heading for bio-compatible electrodes [29]. The direct electropolymerization of ionically modified monomers was for example carried out by Reynolds et al. for sulfonic acid functionalized pyrrole [30–31]. The groups of Heeger et al. [32–34], Bäuerle et al. [35] and Visy et al. [36] synthesized sulfonic acid and carboxylic acid functionalized polythiophenes to study the so called “self-doping” effect of conducting polymers [37]. Interwoven polymeric composite materials based on polymer blends were obtained by electrodepositing sulfonic acid modified bithiophene followed by bipyrrole monomers [38].

In some cases the direct polymerization of ionically modified monomers remains problematic: this was for example reported in the case of sulfonic acid modified pyrrole, where film deposition was only possible when a copolymerization with pristine pyrrole was conducted [30].

Post-polymerization processes on the other hand have to provide high yields and mild reaction conditions to keep the formed polymer backbone intact and to reach a considerable degree of conversion of functional groups. The Cu(I)-mediated 1,3-dipolar cycloaddition between azides and alkynes (“click”-reaction) is a commonly used reaction in post-polymerization processes [39–40]. In the case of the azidomethyl-modified EDOT (EDOT-N_3_) building block different approaches of modifying the corresponding polymer PEDOT-N_3_ have been conducted so far, including the modification of electropolymerized PEDOT-N_3_ with different redox functionalities as employed by Bäuerle et al. [41–43]. The PEDOT relative, propargyl-substituted chemically synthesized 3,4-propylenedioxythiophene was used by Kumar et al. to introduce ionic groups by “click”-chemistry to render the solubility of the gained polymers from organic solvents to water solubility [44]. “Electro-click” modifications were used for chemically synthesized PEDOT-N_3_ and copolymers of EDOT-N_3_ and EDOT with halogens and fluorescent markers [45–46] and of electrochemically synthesized PEDOT-N_3_ to introduce fluorinated alkyl chains [47]. In the latter case the water contact angles could be gradually varied.

We here present the electrocopolymerization of EDOT-N_3_ with the branched terthiophene 2,2’:3’,2’’-terthiophene and the post-polymerization into an ionically modified copolymer. Only recently, we reported on the copolymerization of 3T and EDOT as a straightforward approach to conducting polymer films with tailored HOMO levels and therefore band gap values by varying the monomer ratio during chemical (with FeCl_3_ as oxidant) and electrochemical polymerization [24]. Characterization of the chemically polymerized copolymers by ^1^H DOSY NMR and MALDI–TOF spectroscopy indicated that the EDOT and 3T units are covalently linked. While comparisons with these chemically synthesized polymers support our finding, a real proof of copolymer formation for the electropolymerized films was still missing in our previous publication. Here, we show that by comparison of blend films of the homopolymers with copolymer films obtained by electropolymerizing monomer mixtures the copolymer formation, i.e., the covalent linkage of the two co-monomers can be proven also for the electrochemically synthesized films by means of electrochemical and spectroscopic (in situ and ex situ) techniques.

We further show that the redox properties of the polymers remain identical when EDOT–N_3_ is used as a co-monomer instead of EDOT and that “click”-chemistry is a versatile tool to largely modify material properties, e.g., by the introduction of covalently bound ionic groups.

## Results and Discussion

**Scheme 1:**
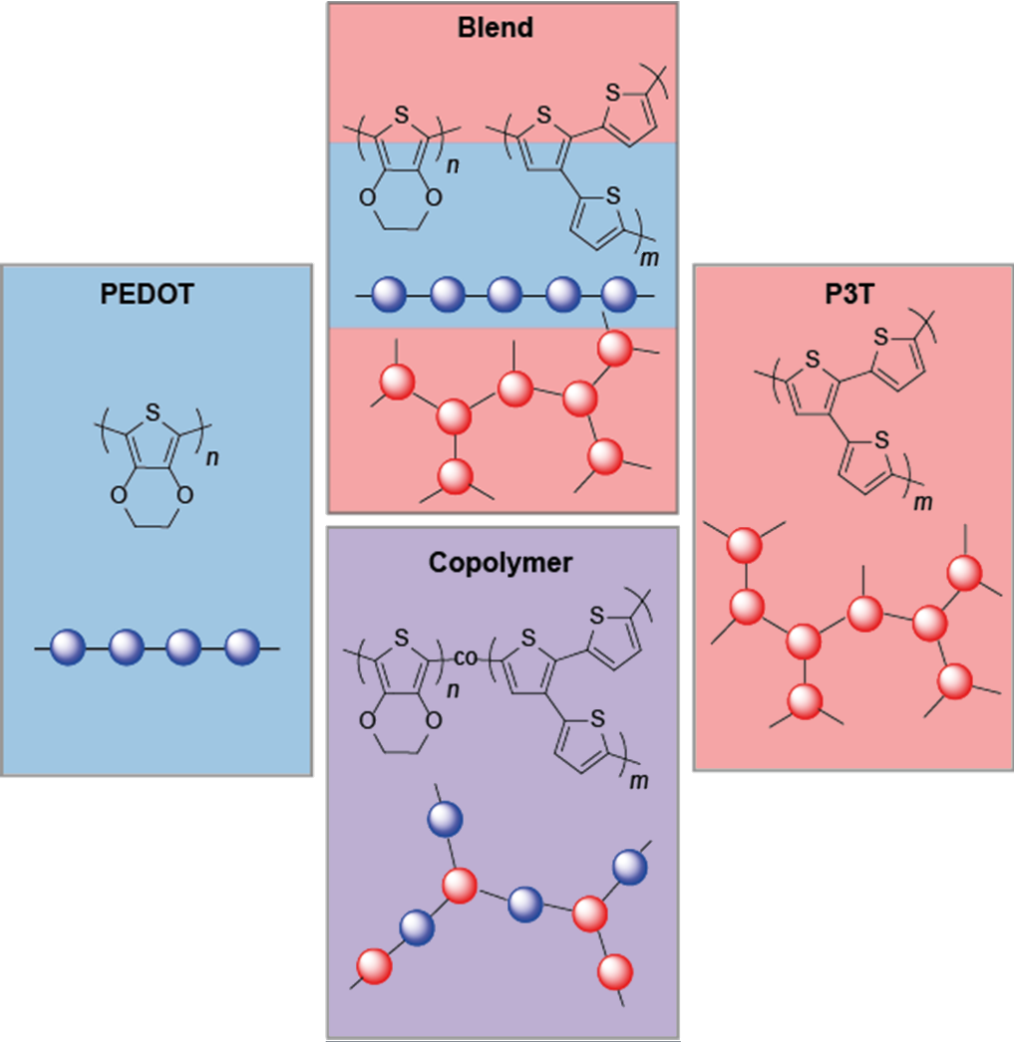
Polymer constitutions of the electropolymerized films.

Electropolymerization of monomer mixtures does not necessarily lead to copolymers of the two monomers but can also result in polymer blend structures. This is often a difficult task to prove [48]. Case one is that both monomers readily react with each other and the polymerization of a mixture of monomers leads to a copolymer. In case two, both monomers react with themselves but not with each other and the polymerization of the monomer mixture leads to a mixture of the two homopolymers which can be regarded as a polymer blend. We mimicked the latter case by consecutive electro-deposition of layers of the respective homopolymers on the electrode. We have shown in earlier work [24] that the oxidation potentials of EDOT and 3T at 1.0 V and 1.1 V vs Fc/Fc^+^, respectively, do principally allow for copolymer formation. Scheme 1 highlights the different polymer constitutions of the electropolymerized films we discuss in the present article: pure homopolymer films P3T and PEDOT, the blend film containing both P3T and PEDOT layers and the copolymer which represents a random combination of the monomers EDOT and 3T and strongly depends on the ratio of the monomers used during the electropolymerization. The nomenclature of the copolymers is in accordance to our previous publication [24]: P(EDOT-*co*-3T)-1:1 means for example that a 1:1 mixture of 3T and EDOT is used during polymerization.

**Figure 1:**
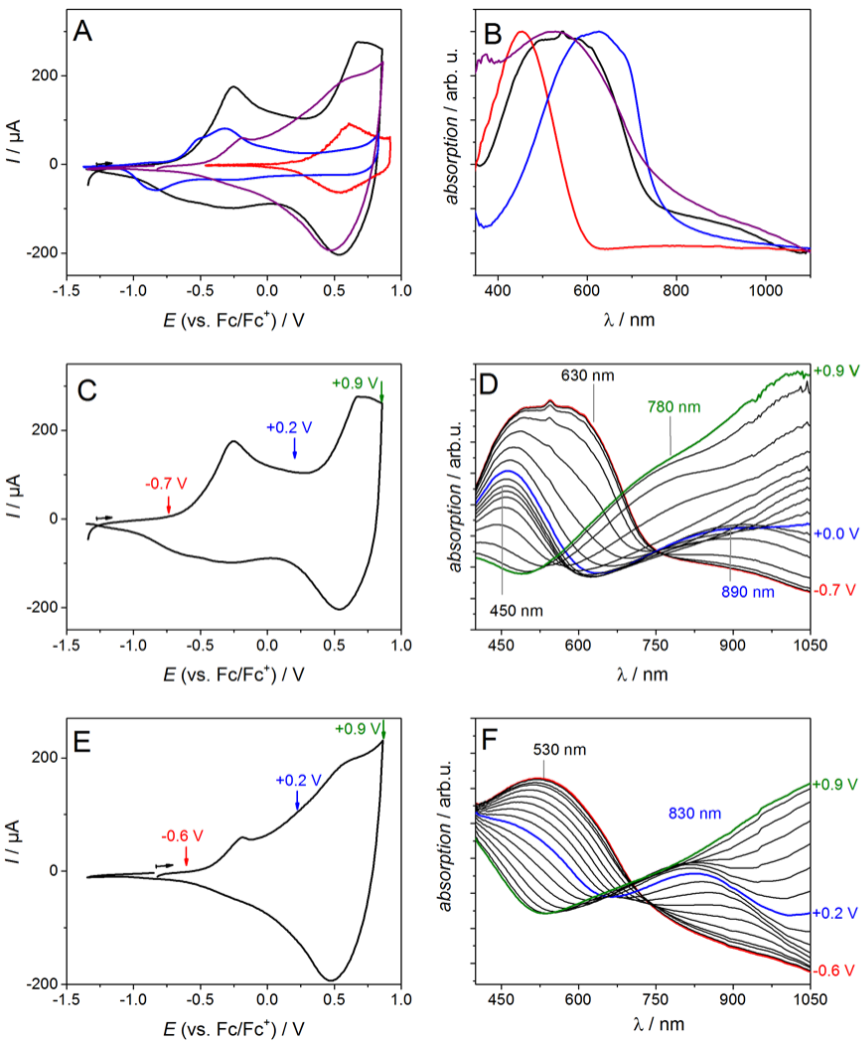
Cyclic voltammograms in 0.1 M NBu_4_PF_6_/MeCN (A) and vis–NIR spectra (B) of electropolymerized films of PEDOT (blue curve), P3T (red curve), PEDOT/P3T-blend (black curve) and P(EDOT-*co*-3T)-1:1 (purple curve) deposited on ITO-coated glass substrates. C–F: In situ spectroelectrochemistry of films deposited under potentiostatic control on ITO with oxidative cycles at 50 mV s^−1^ in 0.1 M NBu_4_PF_6_/MeCN (C and E) and vis–NIR spectra (D and F) recorded during the forward scan in the oxidation process of PEDOT/P3T-blend (C and D) and P(EDOT-*co*-3T)-1:1 (E and F). The films were prepared according to [24].

Figure 1A summarizes representative cyclic voltammograms (CVs) of PEDOT, P3T, a PEDOT/P3T-blend and the copolymer P(EDOT-*co*-3T)-1:1. During the electrochemical oxidation the homopolymers PEDOT and P3T both show chemically reversible behavior, but they differ significantly in their onset potentials of −0.8 V and 0.3 V vs Fc/Fc^+^, respectively (blue and red curves). The cyclic voltammogram (CV) of the PEDOT/P3T-blend has two current maxima located at −0.3 and +0.7 V. Both values correspond to the oxidation potentials of the respective homopolymers indicating the combination of the redox properties of PEDOT and P3T. The P(EDOT-*co*-3T)-1:1 film on the other hand shows one broad oxidation wave with an onset potential of −0.6 V. The UV–vis absorption spectra of the neutral electropolymerized films are shown in Figure 1B: while the homopolymers exhibit absorption maxima at 630 nm for PEDOT and 450 nm for P3T, the PEDOT/P3T-blend and the copolymer P(EDOT-*co*-3T)-1:1 film show rather broad spectral shapes with maxima around 530 nm. From the spectra of the neutral compounds a clear distinction between the copolymer and the blend is not possible.

Monitoring the optical properties during the electrochemical oxidation process by in situ spectroelectrochemistry, however, gives further information about the electronic properties of the charged species and thus allows to allocate redox states and absorption bands to certain species. The recorded spectra of the blend and the copolymer films reveal remarkable differences. Figure 1C and D show the CVs and the absorption spectra recorded during the forward scan of the PEDOT/P3T-blend film. Following the process of the first oxidation wave in the CV the broad absorption band is decreasing asymmetrically upon potential increase. The loss of the shoulder of the absorption band at around 630 nm suggests that PEDOT is oxidized first yielding the charged PEDOT species with an absorption of the radical cation around 890 nm. This is in accordance to literature where the PEDOT radical cation is described with an absorption maximum around 880 nm [24]. Only when approaching the second oxidation wave around +0.9 V the absorption band at 450 nm is decreasing, revealing a new absorption at 780 nm which can be attributed to the radical cation formation of P3T matching the literature value [24]. To our knowledge this is one of very few examples [48–49], where a polymer blend provides the separated absorption and redox properties of the homopolymers which allow for the separated addressing of the polymers by CV and monitoring thereof by spectroscopy.

The copolymer P(EDOT-*co*-3T)-1:1 (Figure 1E and F) shows, as described above, one broad oxidation wave in the CV and a broad absorption with a maximum at 530 nm. During the oxidation the 530 nm band is decreasing uniformly and steadily while at 830 nm a single band is ascending, which indicates the formation of the charged radical cation species. This is in agreement with our earlier data where we showed this uniform steady decrease of the band at 830 nm absorption during the oxidation for P(EDOT-*co*-3T) polymer films with different ratios of the monomers EDOT and 3T [24]. This, with respect to the blend films, completely opposite behavior is a reliable argument that indeed a copolymer is formed from the copolymerization of EDOT and 3T. Table 1 summarizes the characteristic values for the neutral and charged polymer films.

**Table 1:** Summary of absorption and electrochemical characteristics of polymer films electrochemically deposited on ITO electrodes under potentiostatic control derived from in situ spectroelectrochemical experiments in 0.1 M NBu_4_PF_6_/MeCN.

Polymer film	*E*^ox^_onset_ [V vs. Fc/Fc^+^] (HOMO [eV])^a^	λ_max_ (neutral) [nm]^b^	λ_max_ (radical cation) [nm]^b,c^

P3T^d^	+0.3 (-5.4)	450	780
PEDOT^d^	−0.8 (−4.3)	630	880
PEDOT/P3T-blend	−0.7 (−4.4)	–	≈780 and ≈890
P(EDOT*-co-*3T)-1:1	−0.6 (−4.5)	530	830

^a^HOMO levels calculated using −5.1 eV as formal potential of the ferrocene/ferrocenium (Fc/Fc^+^) redox couple in the Fermi scale [50]; ^b^the error is estimated to be ±15 nm; ^c^values determined at *E* = *E*^ox^_onset_ +0.5 V as previously described in [24]; ^d^from [24].

**Figure 2:**
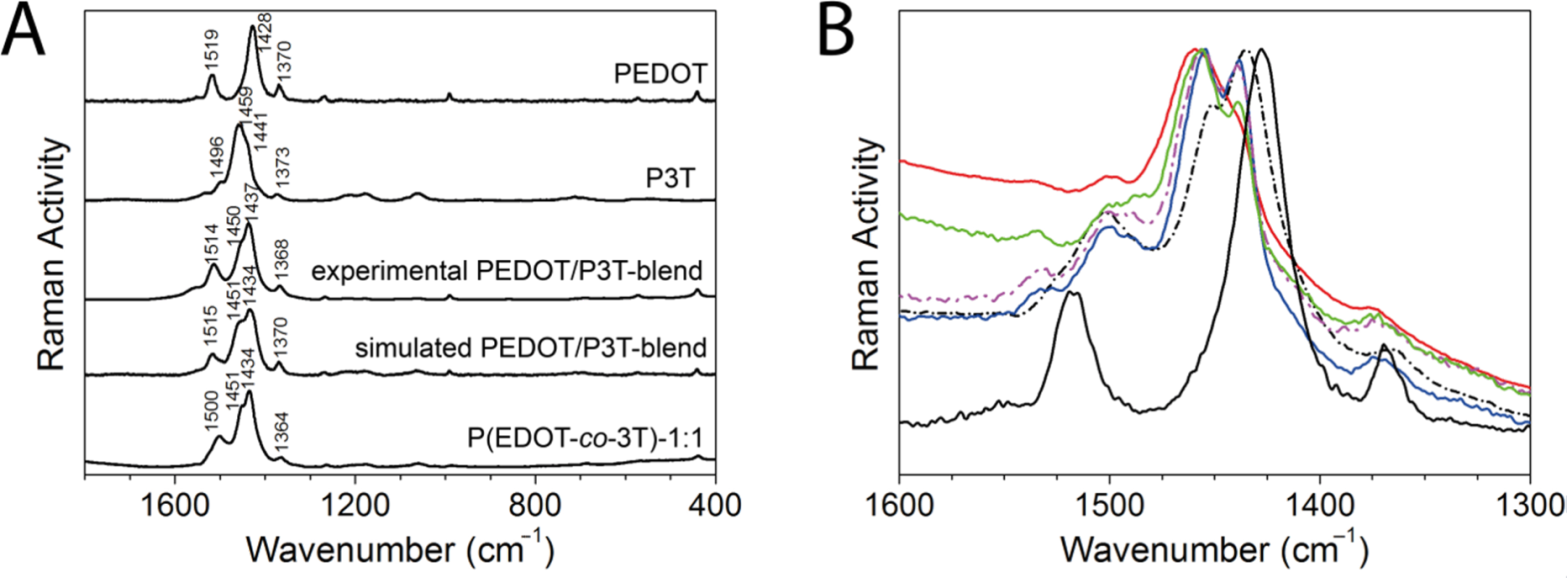
A: Raman spectra of electrodeposited films of the homopolymer P3T and PEDOT, PEDOT/P3T-blend, a simulated spectrum of a blend of PEDOT and P3T (created by adding the spectra of the two homopolymers) and the copolymer P(EDOT-*co*-3T)-1:1, λ_exc_ = 532 nm. B: Comparison of Raman spectra between 1300 and 1600 cm^−1^ for different monomer ratios of the copolymer films (black curve EDOT, broken black curve 1:1, blue 1:3, broken pink curve 1:5, green curve 1:10, red curve P3T), λ_exc_ = 532 nm.

As a further analytical tool we employed Raman spectroscopy which addresses the different vibrational modes of the samples. Figure 2A shows the Raman spectra of the homopolymers PEDOT and P3T, the blend film PEDOT/P3T-blend and the copolymer P(EDOT-*co*-3T)-1:1. Note that the spectra are recorded at 532 nm in order to be in resonance with the lowest energy absorption band (HOMO–LUMO transition) of the neutral polymers and therefore out-of-resonance with the absorption bands of the oxidized species. In accordance with earlier work [51], the Raman spectrum of PEDOT exhibits a very intense band at 1428 cm^−1^ which is associated with a symmetric C_α_=C_β_ stretching and two less intense bands at 1519 and 1370 cm^−1^ which arise from asymmetric C_α_=C_β_ and C_β_–C_β_ stretching vibrations, respectively. In the branched P3T polymer, a broadening of the band associated with the collective C_α_=C_β_ stretching mode (at 1459 cm^−1^) is observed with a shoulder appearing at 1441 cm^−1^ while the two less intense bands appear at 1496 and 1373 cm^−1^. The broadening of the spectra in P3T is related to its branched architecture with different conjugation paths along their π-conjugated backbones which also results in a lowering of the molecular symmetry and an increase of molecular flexibility when compared to the linear polymers.

On the other hand, the Raman spectrum of the blend is clearly a simple superposition of the homopolymer spectra. Note the very good correlation between the spectral profiles of the experimental blend and the calculated spectrum created by adding the spectra of the two homopolymers PEDOT and P3T in Figure 2A. A direct comparison between the spectra of the blend and copolymer, however, reveals a noticeable downshift of the asymmetric C_α_=C_β_ stretching modes (from 1514 in PEDOT/P3T-blend to 1500 cm^−1^ in P(EDOT*-co-*3T)-1:1) and the C_β_–C_β_ Raman bands (from 1368 in PEDOT/P3T-blend to 1364 cm^−1^ in P(EDOT*-co-*3T)-1:1). This frequency downshift strongly suggests that the copolymer has an improved π-conjugation because of the better π-electron delocalization through the covalently connected 3T and EDOT units. A detailed superposition of the spectra of the blend and the copolymer (see labelled bands in Figure S1 in Supporting Information File 1), evidences the presence of two new bands in the copolymer at 1204 and 1060 cm^−1^ which can be assigned to stretchings of newly formed C_α_–C_α_ bonds [52] between the monomers EDOT and 3T and to C_β_–H bending modes [51], respectively. This gives further evidence that the materials formed are copolymers rather than polymer blends.

The Raman spectra also give valuable information about different compositions in the copolymers. In a similar manner as described in our earlier work [24] we prepared copolymer films with different compositions (ratio of the monomers EDOT and 3T during polymerization ranging from 1:1 to 1:10). Figure 2B shows the Raman spectra between 1300 and 1600 cm^−1^ of the copolymers as well as of the homopolymers P3T and PEDOT for comparison. The main Raman bands of all copolymers which are associated with the collective symmetric C_α_=C_β_ stretching modes are located between the band maxima in P3T (1459 cm^−1^) and PEDOT (1428 cm^−1^) and downshift from 1456 to 1435 cm^−1^ on passing from P(EDOT-*co*-3T)-1:10 to -1:1. It is well established in literature that the frequency of this band shifts downward upon increasing conjugation length or increasing quinoidization [53–54]. Therefore, the shift towards lower frequencies (i.e., lower energies) with increasing EDOT content demonstrates that the incorporation of EDOT in a branched thiophene polymer improves the conjugation length. This can be ascribed to the significant participation of the oxygen atoms in the π-conjugation and a gain in rigidity of the polymer backbone due to intramolecular sulfur–oxygen interactions [55–56]. The asymmetric C_α_=C_β_ stretching modes upshift and increase in intensity with increasing EDOT content while the C_β_–C_β_ mode slightly increases in intensity; this is also in accordance with an improved effective conjugation in going from P(EDOT-*co*-3T)-1:10 to -1:1. Note that a similar spectral evolution is found when the sample is recorded with different excitation wavelengths (see Figure S2 in Supporting Information File 1).

### Further functionalization

Summarizing the first part of our manuscript the electropolymerization of monomeric mixtures of 3T and EDOT leads to copolymers and the HOMO levels of these polymers are adjustable by varying the ratio of the monomers during the polymerization process. In a next step we further transferred the copolymerization approach to the azidomethyl-substituted EDOT-N_3_ monomer which allows for the straightforward modification with various alkynes by Cu(I)-catalyzed 1,3-dipolar cycloaddition (“click”-reaction) [57].

**Figure 3:**
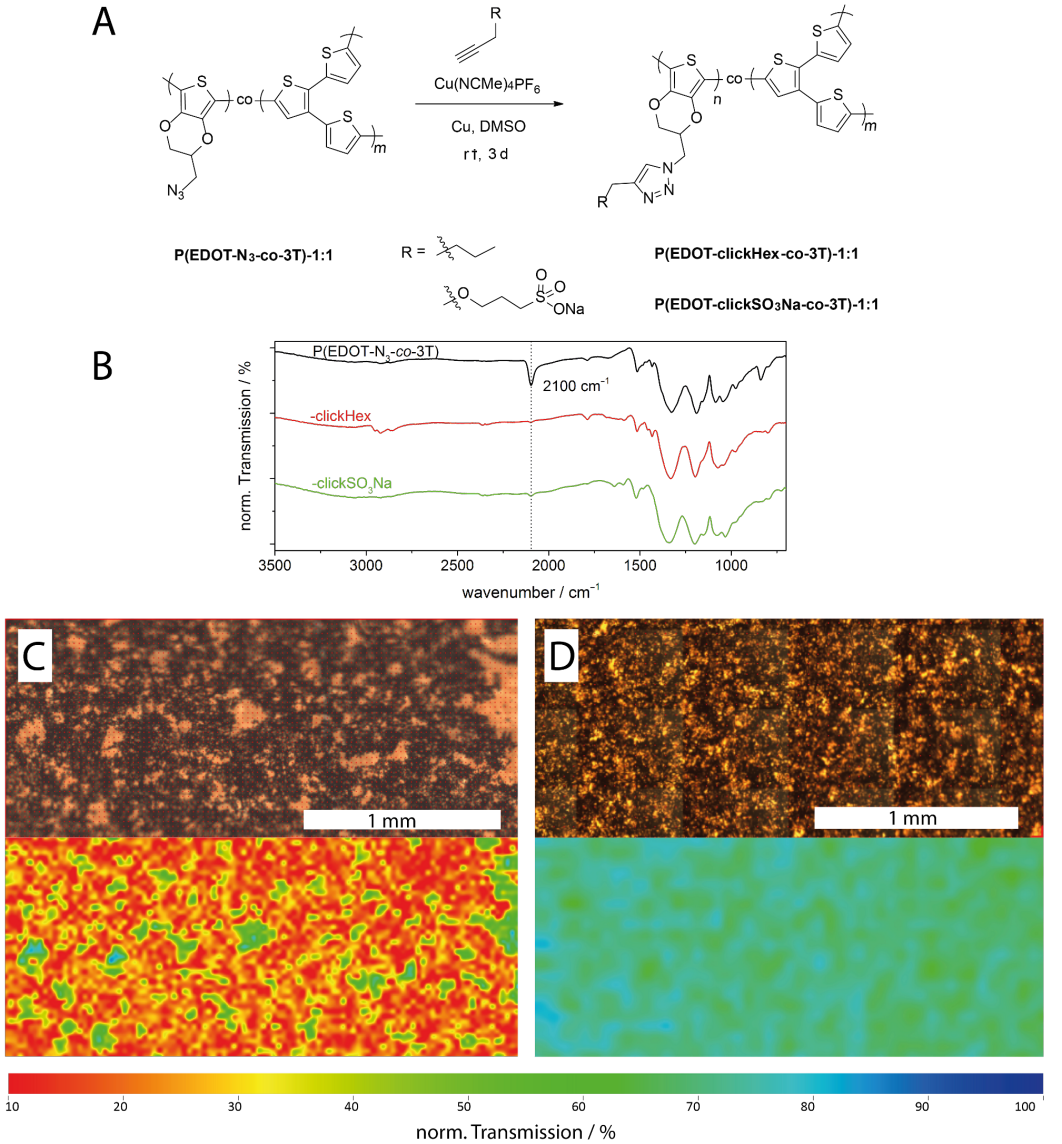
A: Modification of the copolymer films P(EDOT-N_3_*-co-*3T) with 1-hexyne and alkyne sulfonate with [Cu(NCMe)_4_]PF_6_ and copper powder as catalyst in DMSO, reaction time 3 days at room temperature (rt). B: IR spectra of copolymer films P(EDOT-N_3_*-co-*3T) (black line), P(EDOT-clickHex*-co-*3T)-1:1 (red line, hexyl modification) and P(EDOT-clickSO_3_Na*-co-*3T)-1:1 (green line, sulfonate modification); spectra were rescaled (y-offset) for comparison reasons. C, D: Microscope images (top) and IR mappings (bottom) of the azide band intensity at 2100 cm^−1^ of copolymer films deposited under potentiostatic control on gold in 0.1 M NBu_4_PF_6_/MeCN. P(EDOT-N_3_*-co-*3T)-1:1 (C, before modification) and P(EDOT-clickSO_3_Na*-co-*3T) (D, after sulfonate modification). Spectra of the IR microscopy were normalized and plotted with identical colour ranges for comparison reasons.

For the functionalization experiments we chose the copolymer prepared with the highest EDOT-N_3_ content namely the 1:1-copolymer. The functionalization was first conducted with 1-hexyne as a model system. The successful modification had been displayed earlier by Bäuerle et al. who could show, that the cycloaddition takes place with high conversion rates both with EDOT-N_3_ and when the reaction was performed in electropolymerized films of PEDOT-N_3_ [41]. As functional moiety we introduced an ionic alkyne sulfonate (SO_3_Na–alkyne). As the cycloaddition-reaction with SO_3_Na–alkyne is not known in literature we first made tests on the reaction with the monomer EDOT-N_3_ and obtained the product EDOT-clickSO_3_Na in high yield. For ^1^H NMR and IR-spectra we refer to Figure S3 and Figure S4 in Supporting Information File 1. Figure 3A depicts the synthesis of the cycloaddition-reactions of the copolymer films P(EDOT-N_3_*-co-*3T)-1:1 with 1-hexyne and SO_3_Na-alkyne (yielding a butyl end group or a sulfonate end group, respectively). DMSO was chosen as solvent as the degree of swelling is very high [58] and allows modification of the bulk of the film and not just of the surface.

The modified films were analyzed by IR spectroscopy in a combined reflection/absorption mode (RAS) (Figure 3B). The disappearance of the characteristic azide-band at 2100 cm^−1^ indicates that the cycloaddition-reaction takes place almost quantitatively (within the detection limit of IR spectroscopy). For the butyl-modified film P(EDOT-clickHex*-co-*3T)-1:1 (red line) one can also observe new sp^3^-C–H-vibrations at 2800–3000 cm^−1^, which also confirm the successful incorporation of the alkyl moieties. The characteristic bands of the sulfonic acid at 1190 and 1030 cm^−1^ (see Figure S4 in Supporting Information File 1) overlap with other bands of the polymer P(EDOT-clickSO_3_Na*-co-*3T) (green line) in the fingerprint region and therfore a proper assignment is difficult. Further information was gained by a mapping of the polymer films with IR-microspectroscopic measurements. Figure 3 shows the microscope images of the polymer films P(EDOT-N_3_*-co-*3T)-1:1 (C, top, before modification) and P(EDOT-clickSO_3_Na*-co-*3T)-1:1 (D, top, after sulfonate-modification) and the corresponding maps (bottom) of the intensity of the azide-band at 2100 cm^−1^ wherein the highest azide band intensities (transmission of 10%) are displayed in red, and the lowest intensities (transmission of 100%) are displayed in blue. For comparison and to limit the influence of the film thickness all transmission spectra were normalized. While for P(EDOT-N_3_*-co-*3T)-1:1 the high intensity and a homogeneous distribution of the azide band is observed for the whole film unless the bare gold electrode is visible (light areas in the microscope images; green and blue spots in the IR map. Note that the background of the single IR spectra is about 70–80%), for the modified P(EDOT-clickSO_3_Na*-co-*3T)-1:1 nearly no azide band intensity can be observed. This provides information about the integrity of the modification over the whole film.

**Figure 4:**
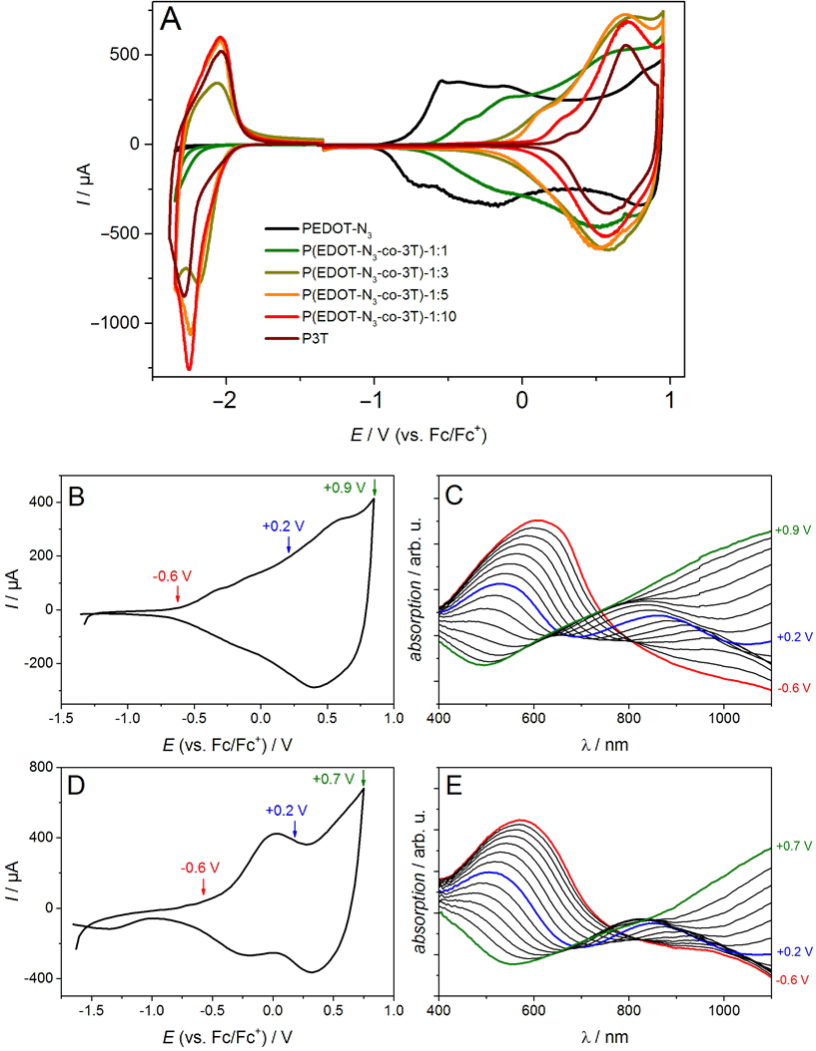
Cyclic voltammograms of films deposited under potentiostatic control on gold-coated glass substrates (1 cm^2^) from different monomer mixtures: (A) Ratio of EDOT-N_3_:3T = 1:0 (black), 1:1 (dark green), 1:3 (light green), 1:5 (orange), 1:10 (red) and 0:1 (brown curve) recorded in 0.1 M NBu_4_PF_6_/MeCN, 20 mV/s. B–E: In situ spectroelectrochemistry of films deposited under potentiostatic control on ITO in 0.1 M NBu_4_PF_6_/MeCN. Cyclic voltammograms (B and D) and corresponding vis–NIR spectra (C and E) recorded during the forward scan of the oxidation of the P(EDOT-N_3_*-co-*3T)-1:1 (B and C) and P(EDOT-clickSO_3_Na*-co-*3T)-1:1 (D and E) at 50 mV s^−1^.

The integrity of the redox and optical behavior upon modification of the P(EDOT-N_3_*-co-*3T)-1:1 with sulfonic acid with regard to the parent copolymer was further proven by cyclic voltammetry and in situ spectroelectrochemistry. It was recently demonstrated that the azide group does not change the oxidation potentials and that the redox behavior of EDOT-N_3_ resembles pristine EDOT [41]. The copolymerization of mixtures of the monomers EDOT-N_3_ and 3T in different ratios (1:1, 1:3, 1:5 and 1:10) was performed in analogy to the copolymerization of EDOT and 3T. The cyclic voltammograms of the respective copolymers show broad oxidation waves with onset potentials ranging in between the onset potentials of the homopolymers P3T and PEDOT-N_3_ (see Figure 4A). The cathodic cycles of the copolymers reveal identical onset potential values and therefore LUMO levels for P3T and the P3T-rich copolymers (1:3, 1:5 and 1:10), while PEDOT-N_3_ and the PEDOT-N_3_-rich 1:1 copolymers show a chemically irreversible electron transfer reaction under our conditions. These data are consistent with those obtained for P(EDOT-*co*-3T) films.

The in situ spectroelectrochemistry data in Figure 4B–E reveals that the ionic modification with sulfonic acid has no influence on the oxidation onset and therefore HOMO level of the polymer film. We attribute this finding to the absence of conjugation between the attached N_3_ groups and the π-system of the polymer backbone. The absorption development upon electrochemical charging shows the characteristic steady decrease of the neutral band and the increase of the absorption at lower energy accounting for the generation of delocalized charges in both the parent polymer and the ionically modified one. The preparation of a conjugated polyelectrolyte with tunable HOMO level is therefore accessible via this modification process.

**Figure 5:**
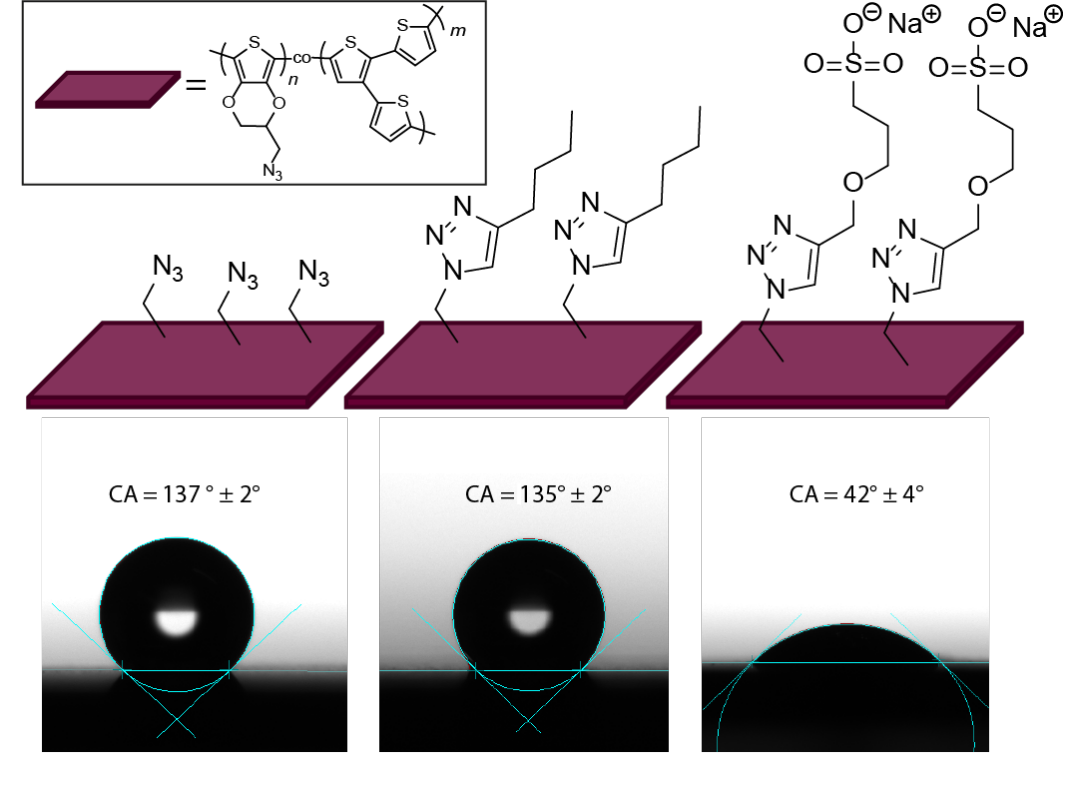
Water contact angle (CA) of the films of P(EDOT-N_3_*-co-*3T) (left), P(EDOT-clickHex*-co-*3T)-1:1 (middle) and P(EDOT-clickSO_3_Na*-co-*3T)-1:1 (right).

While no changes of the electronic properties were detected between P(EDOT-N_3_*-co-*3T)-1:1 and P(EDOT-clickSO_3_Na*-co-*3T)-1:1, the introduction of ions into the polymers can drastically alter the surface properties. Water contact angle measurements are a convenient tool to study the surface polarity. Interestingly, we found in the context of this study that the water contact angle of as polymerized P(EDOT-N_3_*-co-*3T)-1:1 is quite high with a value of 137 ± 2° (Figure 5, left). Similar values were found for P(EDOT*-co-*3T)-1:1 (137 ± 1°). The corresponding homopolymers PEDOT and PEDOT-N_3_ have lower values with a contact angle of 71 ± 3° and 91 ± 6°, respectively, whereas P3T gives an even larger value of 147 ± 6° (see Figure S5 in Supporting Information File 1). This highly hydrophobic surface of the polymers containing branched 3T units may be explained by a high porosity based on a 3-dimensional growth of these polymer films during the electropolymerization process [59–60].

After butyl modification the hydrophobic character remains constant (135 ± 2° after modification, Figure 5, middle), while we changed the surface polarity of the polymer from highly hydrophobic to hydrophilic in the case of the sulfonate modification. A water contact angle of 42 ± 4° is obtained for P(EDOT-clickSO_3_Na*-co-*3T)-1:1 after modification (Figure 5, right), i.e., 100° lower than in the case of the parent polymer P(EDOT-N_3_*-co-*3T)-1:1. In the case of the analogue modification of PEDOT-N_3_ similar trends were observed (see Figure S6 in Supporting Information File 1).

## Conclusion

We showed that the monomers 3T and EDOT are suited for blend formation by sequential electropolymerization, where both polymers maintain their pristine redox features and are both addressable in electrochemical devices. We used in situ spectroelectrochemistry and Raman spectroscopy to prove that copolymer formation from mixtures of the two monomers takes place and that by variation of the feed ratio polymers with adjustable optical and redox properties are accessible. We successfully transferred the copolymerization route to the functional EDOT derivative EDOT-N_3_. As an example we showed the potential of surface polarity adjustment by the introduction of ionic moieties to the hydrophobic polymer films. A decrease of the water contact angle of ≈100° could be achieved which evoked a complete change of the polymer surface nature from hydrophobic to hydrophilic. This opens the way to a library of polymers which carry tailored redox properties as well as additional functionalities through a manifold of different functionalization possibilities by “click”-chemistry by leaving the redox properties unchanged. Such an orthogonal functionalization may further be used to control the properties in new materials for organic photovoltaics where low oxidation potentials are needed (monomer ratio) and a good processability from environmental solvents (e.g., water) is desired (introduction of polar/ionic groups).

## Experimental

### Materials

All chemicals and solvents were purchased from Sigma-Aldrich or Alfa-Aesar. Solvents were at least of HPLC grade and were used as received except otherwise noted. NBu_4_PF_6_ (Sigma-Aldrich, electrochemical grade) was stored in a desiccator over silica beads (activated in an oven at 80 °C for several days). Acetonitrile (Alfa Aesar, supergradient HPLC grade (far-UV), +99.9%) was stored over neutral Al_2_O_3_ (activated under vacuum at 120 °C for 24 h) under an argon atmosphere. DMSO was distilled in vacuum, crystallized at 4 °C and the mother liquor was removed. After melting of the crystals the crystallization procedure was repeated once. DMSO was stored over 3 Å molecular sieves (activated in an oven at 80 °C for several days) under argon atmosphere. All reactions were carried out under argon atmosphere unless otherwise noted. The synthesis of 3T was published elsewhere [61].

### Methods

^1^H (250 MHz) and ^13^C{^1^H} (63 MHz) NMR spectra were recorded on a Bruker Avance 250 spectrometer. vis-NIR spectra of the polymer films deposited on ITO-coated glass substrates were recorded with a Lambda 35 spectrometer (Bruker).

IR spectroscopy was performed on a Bruker FTIR spectrometer IFS 66/S in a wavelength region of 600–3500 cm^−1^. Liquids and oils were measured on a diamond-ATR-device (golden gate). Polymer films were measured with a VeeMAX^TM^ II Variable Angle Specular Reflectance Accessory (Pike Technologies) with an incidence angle differing 35° from the orthogonal plane. IR-microscopy experiments (ultra-fast mappings) of deposited films were performed with a Thermo Fisher Scientific Nicolet iN10 MX spectrometer equipped with a nitrogen cooled MCT/A detector. The sampling interval was 0.10 s, with a step size of 25 µm or 50 µm. The spectral resolution was 16 cm^−1^, the aperture was set to 80 µm or 150 µm. Spectra are corrected for background. Modified electrodes were directly placed under the microscope. Data acquisition was performed with Thermo Fisher Scientific Omnic Picta software. Raman spectra with 532 laser excitation were recorded using a Bruker Senterra dispersive Raman microscope equipped with a neon lamp and using a Nd:YAG laser with excitation at λ_exc_ = 532 nm. Raman spectra with excitation at λ_exc_ = 488 nm were recorded by using a Microscope Invia ReflexRaman RENISHAW. Fourier Transform (FT) Raman spectra with 1064 nm laser excitation were recorded using a Bruker FRA 106/S instrument and a Nd:YAG laser source with excitation at λ_exc_ = 1064 nm, operating in a back-scattering configuration. Water contact angle measurements were carried out on a Contact Angle System OCA 20 by dataphysics using water droplets of 1–2 µL volume (*R* > 18.2 MΩ). Calculations of the contact angles were done with the software SCA20 using ellipse fitting. Mean values were calculated using at least 4 different measurements. Deviations given are the mean values of al obtained single deviations.

### Electrochemical experiments

All electrochemical experiments were performed with an Autolab PGSTAT101 potentiostat (Metrohm, Germany) in a three-electrode glass cell under argon atmosphere at room temperature. The counter electrode was a Pt plate. The pseudoreference electrode consisted of AgCl-coated silver wire that was directly immersed into the electrolyte. As working electrodes, gold (50 nm layer)-coated Si wafers (with a 5 nm Cr adhesion layer between the Si wafer and the Au layer) or ITO-coated glass (≤50 Ω/sq, PGO, Germany) slides (approximately 1 cm^2^) were used. The gold working electrodes were fabricated by the physical vapor deposition of Cr and Au on rotating Si wafers. The gold-coated Si wafers and the ITO substrates were thoroughly washed with acetone prior to use. Additionally, the electrodes were treated with oxygen plasma for at least 5 min before electrodepositions. NBu_4_PF_6_ was used as the supporting electrolyte at a concentration of 0.1 M. Electrolyte solutions were deaerated by argon bubbling. All potentials are referenced to the formal potential of the Fc/Fc^+^ external redox standard [62]. To avoid charge-trapping effects during cyclic voltammetric experiments, the oxidation and reduction cycles were performed separately.

Visible–NIR spectroelectrochemical measurements were made in situ with transparent ITO electrodes (on glass, ≤50 Ω/sq, PGO, Germany) and 0.1 M NBu_4_PF_6_/MeCN as the electrolyte. The electrodeposited films were used directly for the measurements. The vis–NIR spectra were recorded with a diode array spectrometer from the Zeiss MCS 600 series (equipped with a Zeiss CLH600 halogen lamp and two MCS 611 NIR 2.2 and MCS 621 VIS II spectrometer cassettes).

**Electropolymerization of PEDOT, PEDOT-N_3_, P3T and copolymers:** In a similar manner as described in reference [24] electropolymerization of the monomers 3T, EDOT and EDOT-N_3_ on gold or ITO was performed under potentiostatic control (deposition time = 200 s) with an overall monomer or comonomer concentration of 2 mM in 0.1 M NBu_4_PF_6_/MeCN at 0.9–1.1 V vs Fc/Fc^+^, followed by a discharging step (200–205 s, −1.4 V vs Fc/Fc^+^). The deposition potentials correspond to the peak potential of the overlapping signal of the oxidation of both comonomers in the corresponding electrolyte. The desired concentrations were achieved by taking appropriate aliquots from stock solutions (*c* = 10 or 20 mM). For blend formation, four consecutive potentiostatic polymerizations (each for 50 s at 0.9–1.1 V vs Fc/Fc^+^, followed by a discharging step of 50 s at −1.4 V vs Fc/Fc^+^) were conducted alternating the used monomer in each step. For this purpose, two identical cells, one loaded with EDOT and one with 3T (*c* = 2 mM) were used. Between the steps, the polymer films were rinsed thoroughly with pure acetonitrile.

### Synthesis

2-(Azidomethyl)-2,3-dihydrothieno[3,4-*b*][1,4]dioxine (PEDOT-N_3_) [42,63] and sodium 3-(prop-2-yn-1-yloxy)propane-1-sulfonate (SO_3_Na–alkyne) [64] were synthesized based on literature.

**Synthesis of sodium 3-((1-((2,3-dihydrothieno[3,4-*b*][1,4]dioxin-2-yl)methyl)-1*H*-1,2,3-triazol-4-yl)methoxy)propane-1-sulfonate (EDOT-clickSO_3_Na):** Sodium 3-(prop-2-yn-1-yloxy)propane-1-sulfonate (SO_3_Na–alkyne, 0.1 mmol, 20.0 mg), 2-(azidomethyl)-2,3-dihydrothieno[3,4-*b*][1,4]dioxine (PEDOT-N_3_, 0.1 mmol, 19.5 mg) and tetrakis(acetonitrile) copper(I) hexafluorophosphate (0.005 mmol, 2.0 mg) were dissolved in DMSO (2.5 mL). Copper powder (0.1 mmol, 6.9 mg) was added and the reaction mixture was stirred for 3 days at room temperature. The reaction mixture was filtered, the filtrate was concentrated in vacuum and poured in methanol which was then decanted. Residual solvent was removed under reduced pressure to yield the raw product as a greenish highly viscous oil (37 mg, 93%). ^1^H NMR (250 MHz, DMSO-*d*_6_) δ 8.13 (s, 1H), 6.61 (s, 2H), 4.66 (m, 3H), 4.47 (s, 2H), 4.31 (m, 1H), 3.95 (m, 1H) 3.48 (t, *J* = 6.6 Hz, 2H), 2.4 (t, *J* = 7.5 Hz, 2H), 1.78 (m, 2H) ppm; ^13^C{^1^H} NMR (63 MHz, DMSO-*d*_6_) δ 141.1, 140.8, 125.1, 100.9, 100.6, 72.2, 69.5, 65.7, 63.7, 50.35, 49.6, 26.1 ppm; IR (ATR): 3107, 3006, 2916, 2870, 2484, 2424, 1192, 1035 cm^−1^.

**Synthesis of P(EDOT-clickHex-co-3T) and P(EDOT-clickSO_3_Na-co-3T):** For the polymer analogue “click”-modification of P(EDOT-N_3_*-co-*3T) polymer films on gold or ITO electrodes were placed in flasks containing DMSO (10 mL), tetrakis(acetonitrile) copper(I) hexafluorophosphate (0.005 mmol, 1.9 mg) and copper powder (0.1 mmol, 6.4 mg). Alkyne (sodium 3-(prop-2-yn-1-yloxy)propane-1-sulfonate, 0.1 mmol, 20.0 mg or 1-hexyne, 0.1 mmol, 8.2 mg) was added. The films were allowed to react for three days, while the solution was gently agitated from time to time. The films were thoroughly rinsed with DMSO (2×) and methanol (3×) and dried in vacuum. The success of the reaction was confirmed by IR spectroscopy.

## Supporting Information

File 1 Additional Raman data of PEDOT, P3T, copolymers and blends; ^1^H NMR and IR spectra of EDOT-ClickSO_3_Na; contact angles of P3T; PEDOT-N_3_, PEDOT-clickHex and PEDOT-clickSO_3_Na.
