# Supplementary material for: Functionalized branched EDOT-terthiophene copolymer films by electropolymerization and post-polymerization “click”-reactions
Source: Beilstein J Org Chem. 2015 Mar 11;11:335–47. doi: 10.3762/bjoc.11.39 (PMC4362086; doi:10.3762/bjoc.11.39)
Supplement: File 1 — Additional Raman data of PEDOT, P3T, copolymers and blends; 1H NMR and IR spectra of EDOT-ClickSO3Na; contact angles of P3T; PEDOT-N3, PEDOT-clickHex and PEDOT-clickSO3Na. [file Beilstein_J_Org_Chem-11-335-s001.pdf]

Supporting Information

for

Functionalized branched EDOT-terthiophene copolymer films by electropolymerization and post-polymerization “click”-reactions

Miriam Goll[1], Adrian Ruff[1], Erna Muks[1], Felix Goerigk[1], Beatrice Omiecienski[1], Ines Ruff[2,§], Rafael C. González-Cano[3], Juan T. Lopez Navarrete[3], M. Carmen Ruiz Delgado[3], Sabine Ludwigs*[1]

Address: [1]IPOC-Functional Polymers, Institute for Polymer Chemistry, University of Stuttgart, Pfaffenwaldring 55, 70569 Stuttgart, Germany, [2]Thermo Fisher Scientific GmbH, Im Steingrund 4-6, 63303 Dreieich, Germany and [3]Department of Physical Chemistry, University of Málaga, 29071 Málaga, Spain

Email: Sabine Ludwigs - sabine.ludwigs@ipoc.uni-stuttgart.de

*Corresponding author

[§]née Dreiling

Additional Raman data of PEDOT, P3T, copolymers and blends; ^{1}H NMR and IR spectra of EDOT-ClickSO$_3$Na; contact angles of P3T; PEDOT-N$_3$, PEDOT-clickHex and PEDOT-clickSO$_3$Na.

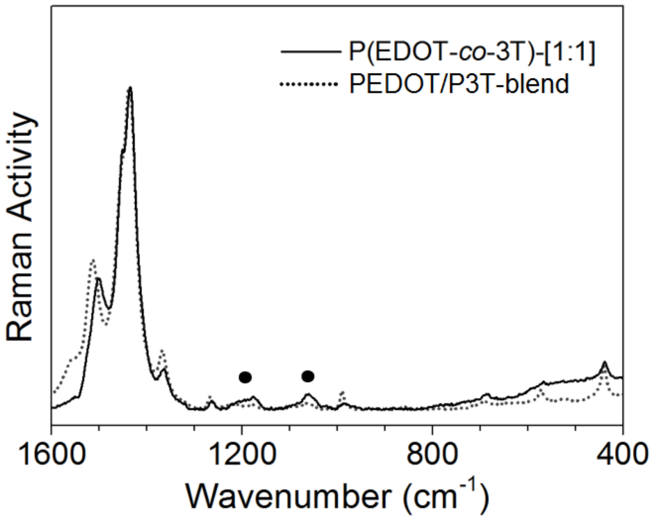

Figure S1: Raman spectra of a blend PEDOT/P3T-blend and copolymer P(EDOT-*co*-3T)-[1:1], recorded at 532 nm.

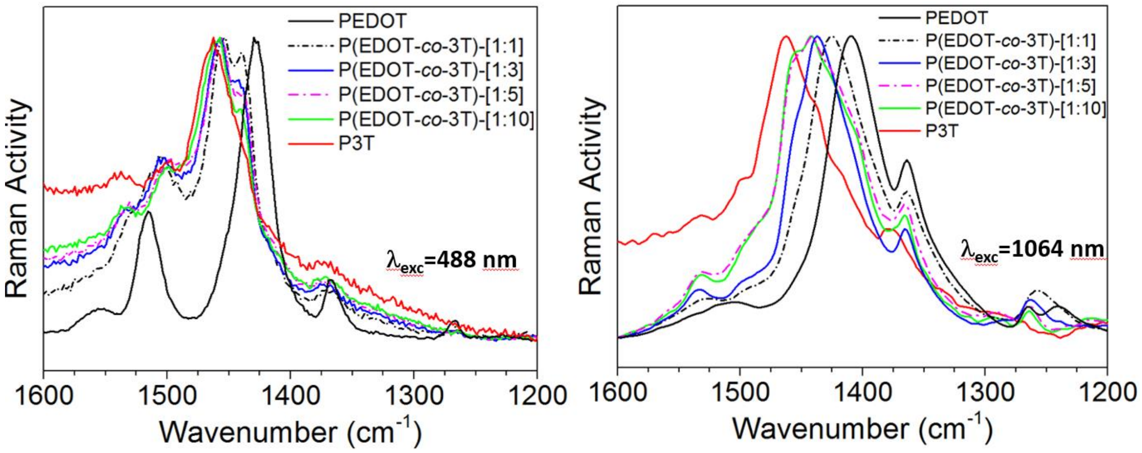

Figure S2: Raman spectra of electrochemically deposited copolymers (potentiostatic control, 0.9-1.1 V) P(EDOT-*co*-3T)-[1:1], -[1:3], -[1:5], and -[1:10] and homopolymers P3T and PEDOT, recorded at 488 and 1064 nm. Note that the spectra recorded at 488 and 1064 nm are in resonance and out-of-resonance Raman conditions, respectively, with the lowest energy absorption band of the neutral polymers. However, the excitation laser excitation at 1064 nm can be in resonance with the absorption band of the charged species what explains the broadening of the spectra, especially in the case of the PEDOT polymer.

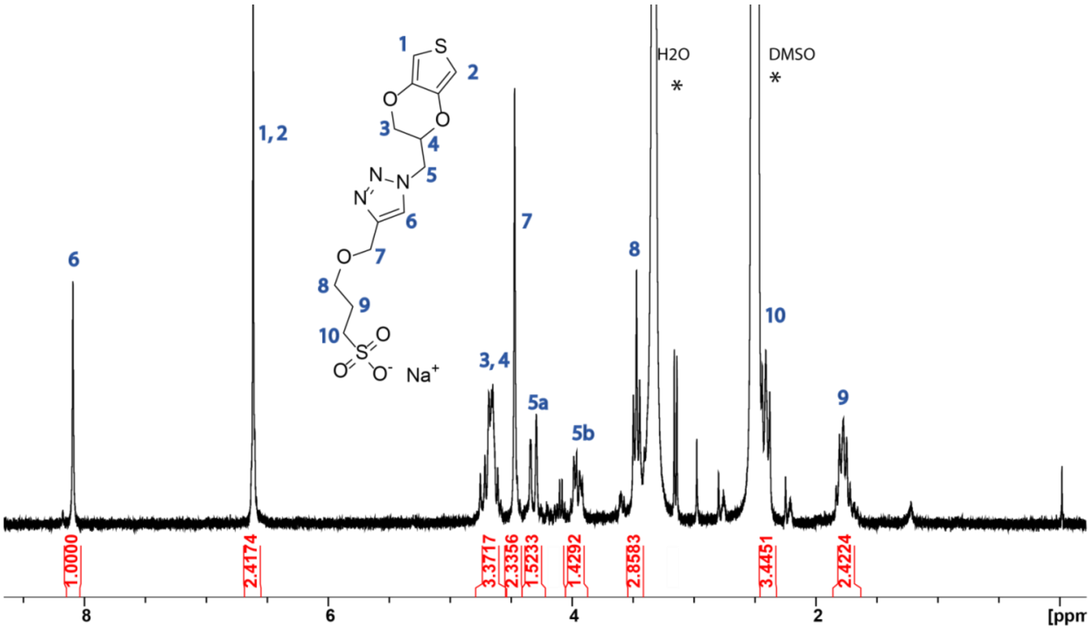

Figure S3: ^{1}H NMR of EDOT-clickSO_3Na, DMSO-d_6

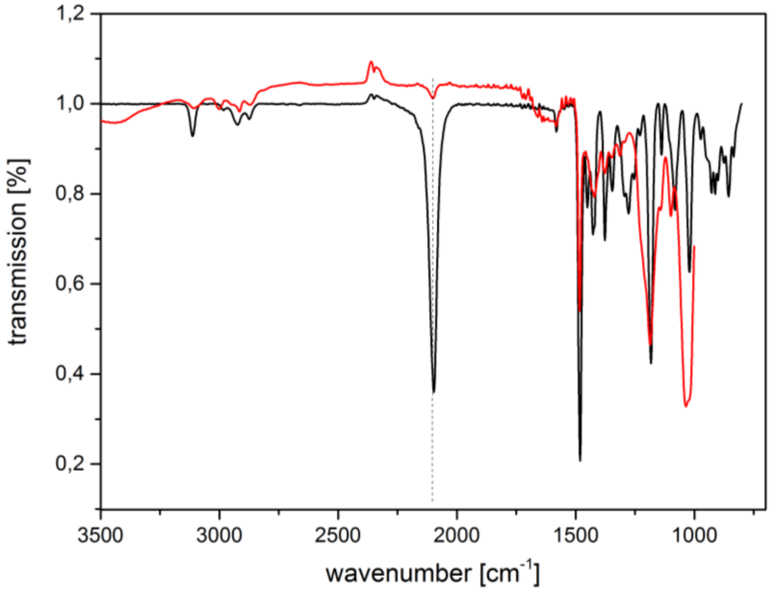

Figure S4: IR spectra of EDOT-N_3 (black) and EDOT-clickSO_3Na (red). Dotted line indicates the position of the azide-band.

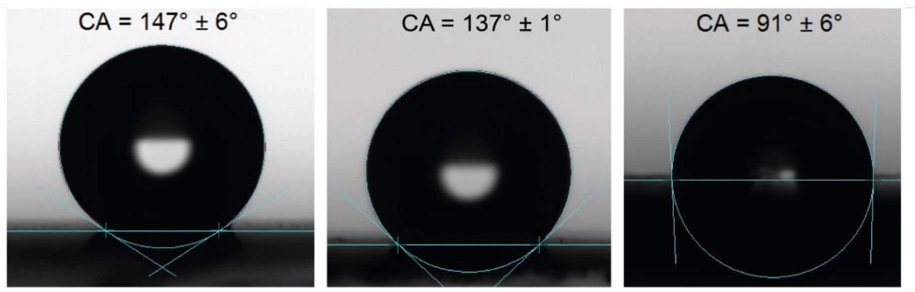

Figure S5: Water contact angles of P3T (left) P(EDOT-*co*-3T)-1:1 (middle), and PEDOT-N_3 (right)

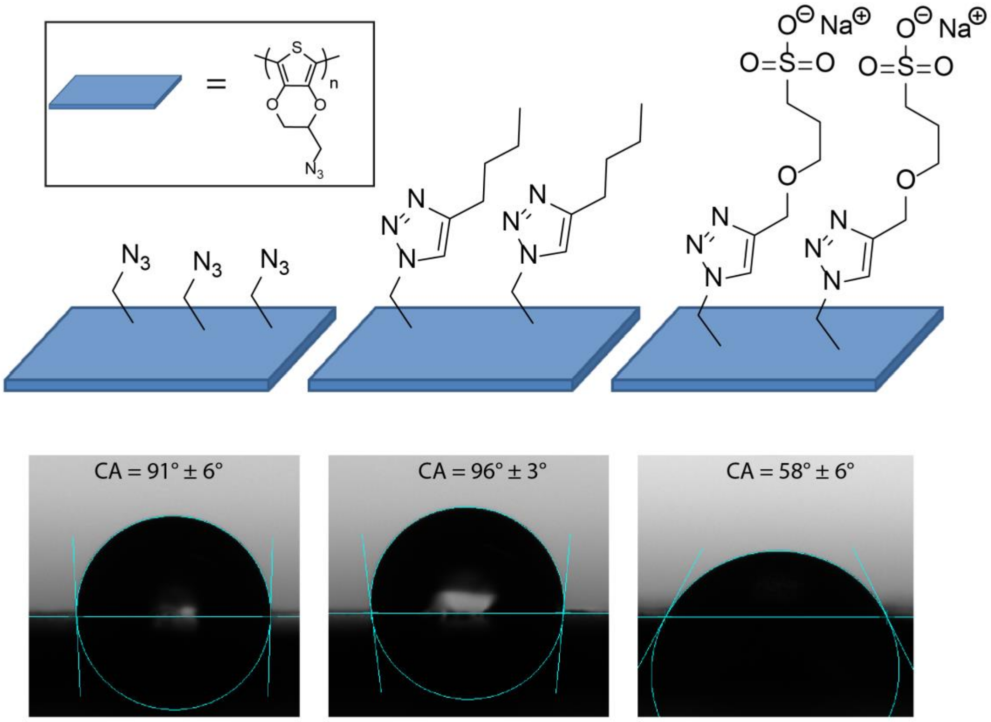

Figure S6: Water contact angle of the films of P(EDOT-N_3) (left), P(EDOT-clickHex) (middle) and P(EDOT-clickSO_3Na) (right).
